# Changes in conditioned pain modulation using anti-Parkinson drugs in patients with Parkinson's disease

**DOI:** 10.1016/j.ensci.2025.100574

**Published:** 2025-07-14

**Authors:** Eiichirou Urasaki, Yasushi Miyagi, Junji Kishimoto

**Affiliations:** a Department of Neurosurgery, Fukuoka Mirai Hospital, Medical Co. LTA (Living Together Association), Fukuoka, Japan; b Center for Clinical and Translational Research, Kyushu University Hospital, Fukuoka, Japan

**Keywords:** Conditioned pain modulation, Cutaneous silent period, Cold pressure, Parkinson's disease, Anti-Parkinson medications

## Abstract

**Objectives:**

Patients with Parkinson's disease (PD) highly complain of pain, probably due to the lowered pain threshold caused by dopamine deficiency. Nonetheless, only a few studies have investigated the effects of anti-PD medications on “pain inhibits pain” ability. This study aimed to evaluate conditioned pain modulation (CPM) using the cutaneous silent period (CSP) and the numerical rating scale (NRS) and to investigate the effect of anti-PD medications on CPM.

**Materials and methods:**

The CSP was recorded in 40 patients with PD under drug-on and drug-off conditions. Changes in the CSP elicited by electrical test stimulation and in the NRS when the patients experienced pain with cold pressure as a conditioned stimulus were assessed. A shortened CSP duration or reduced CSP score due to cold pressure were interpreted as objective CPM responses.

**Results:**

The CSP latency was analyzed in 22 patients when the electromyographic contamination in the CSP waveform was low. The CSP duration shortening during cold pressure was significantly greater under the drug-on condition than under the drug-off condition. The change in CSP duration exhibited a significant correlation with the change in the NRS scores. CSP score analysis was performed on 18 patients in whom latency analysis was difficult owing to electromyographic contamination. In the drug-on state, conditioned cold-pressure pain significantly decreased the CSP score.

**Conclusions:**

Dynamic changes in the CSP caused by cold pressure in patients with PD suggest that anti-PD medications may enhance CPM ability.

## Highlights

• Pain is common in Parkinson's disease, but descending pain inhibition remains underexplored.
• “Pain inhibits pain” reflects a descending inhibitory mechanism testable in humans.
• We used evoked electromyography and pain ratings to assess drug effects on pain control.
• Anti-Parkinson drugs enhanced descending pain inhibition during cold pressure test.
• Drug-related pain relief may differ depending on stage in Parkinson's disease.

## Graphical abstract

**Figure:**
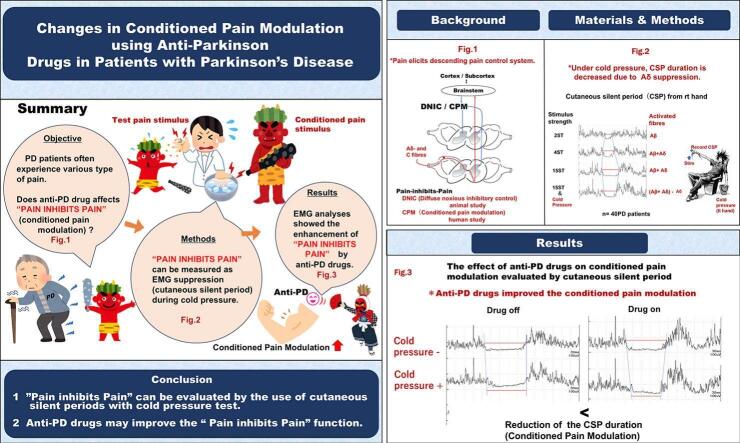
In the cartoon, the little “pain devil” runs away when a bigger “pain devil” shows up. This “pain inhibits pain” effect can be measured with EMG (cutaneous silent period) during a cold pressor test. It shows how the descending pain control system suppresses pain.We found that this effect got stronger after taking an anti‑Parkinsonian drug.

## 1 Introduction

### 1.1 Background

A recent survey indicated that more than two-thirds of patients with Parkinson's disease (PD) suffer from chronic pain [1,2]. Additionally, several studies have demonstrated that patients with PD exhibit increased pain sensitivity. Most of these studies have reported a decreased pain threshold to electrical [3], heat [[3], [4], [5]], or cold [6] stimuli in the dopaminergic medication-off state among patients with PD compared to control subjects; however, the pronociceptive effect of dopaminergic medication to heat stimulation may be seen in predominantly left-sided patients with PD [7]. The nociceptive flexion reflex (also known as the RIII reflex), an objective measure of pain threshold, is generally elicited in patients with PD with a lower pain threshold [3,8,9] than in normal controls, and contradictory results are rare [10].

Two underlying mechanisms have been proposed to explain the increased sensitivity to pain among patients with PD: first, dopamine deficiency may induce pain processing dysfunction in the basal ganglia/cortical areas, which are activated or deactivated by ascending pain signal [11,12], and second, dopamine deficiency reduces the activity of the descending pain inhibitory control system [3]. The role of the basal ganglia in nociception and pain has been extensively studied [12]. Previous reports have shown that medial thalamic activity evoked by noxious electrical stimulation or tail pinch is suppressed by the caudate nucleus and nigral stimulations [11,12] and that complete spinal cord resection does not alter the nigral inhibition of medial thalamic spontaneous activity [11,12], strongly suggesting the presence of a supraspinal pain suppression system, independent of the descending inhibitory interaction. Among the descending pain control networks involving cortical and subcortical regions and brain stem nuclei [13], Le Bars et al. studied the “pain inhibits pain” phenomenon in animal experiments [14] and termed it “diffuse noxious inhibitory control” (DNIC). This phenomenon suggests that pain in one body part (test stimulus) is reduced by pain added to another body part (conditioned stimulus). One of the proposed mechanisms was that the conditioned stimulus signal acts via the subnucleus reticularis dorsalis (SRD) in the lower brainstem to the wide dynamic range (WDR) neuron in the dorsal horn of the whole spinal cord, thereby reducing the pain caused by the test stimulus.[15]

DNIC is assessed by measuring the inhibition of second-order WDR neurons following the application of a noxious stimulus outside the receptive field of the recorded neuron [14,16]. However, in human studies, the “pain inhibits pain” effect is evaluated by examining how the perceived intensity of a noxious test stimulus is modulated by the heterotopic application of another noxious conditioned stimulus [17]. This fundamental methodological difference makes direct comparisons between animal and human studies challenging [17].

Furthermore, many animal studies are conducted under anesthesia, whereas human studies are typically performed on conscious participants [[14], [15], [16], [17]]. Since the presence or absence of consciousness may influence pain modulation mechanisms, it is essential to distinguish between DNIC, which refers to inhibitory effects observed in anesthetized animals, and conditioned pain modulation (CPM), which describes similar effects in conscious humans [17]. This distinction ensures clarity in the interpretation of findings and facilitates appropriate comparisons between experimental models and clinical observations.

Although DNIC has not yet been directly investigated in Parkinsonian animal models, studies employing preclinical models of PD have identified central nervous system regions involved in the modulation of descending pain inhibition [[16], [17], [18]]. These studies have shown that Parkinsonian lesions can disrupt dopaminergic signalling within the periaqueductal grey (PAG), resulting in bilateral alterations in opioid-mediated nociceptive transmission at the level of the dorsal horn of the spinal cord. Additionally, increased excitability of WDR neurons in lamina V has been observed in the spinal cords of rats unilaterally lesioned with 6-hydroxydopamine, which exhibited pronounced mechanical and thermal hypersensitivity [19]. The administration of dopaminergic agents systemically has been reported to restore nociceptive thresholds in rodent models of PD [16].

### 1.2 Study objective

Few studies have been conducted on CPM in patients with PD; nevertheless, they have all proposed that CPM is not involved in pain suppression in PD [3,20,21]. Their CPM results in human clinical research appear to contradict the findings from the aforementioned preclinical literature [16,18,19]. Possible reasons for this discrepancy include differences in assessment methods, differences in the mechanism of CPM and DNIC, or the influence of consciousness [17].

With regard to CPM evaluation methods, various methods are in use, making a unified evaluation difficult. In this connection, the following methods are used. To examine CPM, numerous modalities such as thermal (heat or cold), ischemic, or chemical pain have been used as the conditioned stimulus to inhibit the test stimulus, which is applied as a thermal, mechanical (pressure), electrical, or laser-induced pain stimulus [[22], [23], [24]]. The methods for evaluating the CPM effect vary, ranging from the subjective visual analogue scale and numerical rating scale (NRS) to objective neurophysiological methods, including cerebral evoked potential [24], nociceptive flexion reflex [23], and radiological findings derived from positron emission tomography (PET) [25] or functional magnetic resonance imaging [26,27]. Different testing and assessment methods may yield different results [15]. Furthermore, the process of subjective and objective assessments of changes in pain may differ [15,17,24], necessitating simultaneous comparisons.

This study aimed to analyze subjective NRS scores and objective electro-neurophysiological methods using the cutaneous silent period (CSP) to assess changes in CPM [28] induced by anti-PD medications in patients with PD. We hypothesized that in patients with PD, the improvement in CPM function induced by anti-PD medications could be demonstrated using a novel electrophysiological evaluation method. It was also examined whether the results changed in patients with and without clinical pain.

## 2 Materials and methods

### 2.1 Patients with PD

The clinical characteristics of the patient with PD are presented in Table 1. Data are given as mean (standard deviation). Forty patients with PD (21 women and 19 men) aged 42–72 years [61.9 (7.7)] participated in the study (Table 1). The disease duration, the Mini-Mental State Examination (MMSE), the Levodopa Equivalent Daily Dose (LEDD) and the Unified Parkinson's Disease Rating Scale (UPDRS) part III in 40 patients with PD are shown in Table 1. All patients fulfilled the movement disorder society clinical diagnostic criteria for PD [29]. LEDD was calculated according to Tomlinson et al. [30]. No patients had a history or clinical evidence of dementia, psychiatric disorder, neurological disease other than PD, and other pain conditions, including entrapment peripheral nerve neuropathy, diabetic neuropathy, fibromyalgia, complex regional pain syndrome, or underlying injuries, etc. All patients were screened and examined by a neurologist.

**Table 1:** Patient's characteristics; descriptive statistics and MANOVA results.

	PD total (*n* = 40)	PDN (*n* = 11)	PDP (*n* = 29)	PDN vs PDP (*p* value)
Age (years)	61.9 (7.7)	62.2 (9.4)	61.8 (7.1)	0.853
Disease duration (years)	11.2 (5.9)	11.2 (5.5)	11.2 (6.2)	0.963
MMSE	28.4 (1.8)	28.6 (1.8)	28.4 (1.8)	0.987
LEDD (mg)	936 (434)	1049 (586)	894 (363)	0.320
UPDRS part III (drug on state)	21.8 (14.1)	24.2 (14.3)	20.9 (14.2)	0.519

Data are given as mean (standard deviation).

MMSE, Mini-mental state examination; LEDD, Levodopa equivalent daily dose; PD, Parkinson's disease; PDN, Parkinson's disease without pain; PDP, Parkinson's disease with pain; UPDRS-III, Unified Parkinson's disease Rating Scale Part III.

Twenty-nine patients with PD reported clinical pain related to PD (PDP, Parkinson's disease with pain), while 11 patients (PDN, Parkinson's disease without pain) did not (Table 1). The degree of pain was scored using “sensory complaints related to parkinsonism” in UPDRS part II, section 17, [ranged from 0 (no pain complaints) to 4 (excruciating pain)]. The mean score was 2.2(1.1) in patients with PDP and 0 in patients with PDN.

**Fig. 1:**
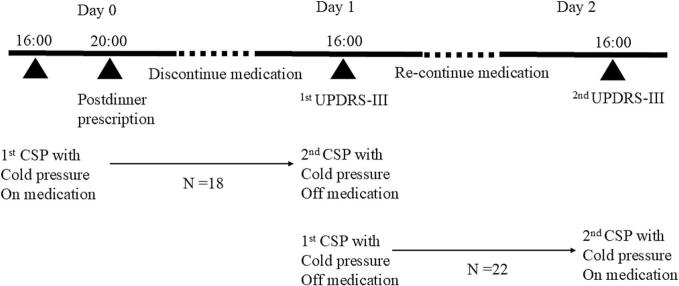
Experimental timeline during medication on/off.

Patients with PD underwent cold pressure testing twice on separate days. The order of the CSP measurements was randomly altered to eliminate the possibility of an order effect (Fig. 1). The test was performed in the “drug-on state” followed by the “drug-off state” in 18 patients and in the “drug-off state” followed by the “drug-on state” in 22 patients. “Drug on state” means when the patient takes anti-PD medications, and “Drug off state” is when the patient stops taking medications. Measurements in the drug-off state were performed at least 20 h after medication withdrawal; anti-PD medications, including long-lasting dopamine agonists, and analgesics, antidepressants, opioids, antineuropathic drugs, neuroleptic drugs were discontinued on the day before the CSP test, following the post-dinner prescription and the CSP in the drug-off state was measured at approximately 4 p.m. on a subsequent day. The CSP in the drug-on state was recorded approximately 24 h before or after the CSP in the drug-off state (Fig. 1).

The patients participating in the study were on several anti-PD medications (Table 2). This fits with the real world but obviously precludes any assessment of the influence of each drug dosage on the parameters under study. Alternatively, LEDD's influence on the study parameters was analyzed (Table 1).

**Table 2:** Medications used in patients with Parkinson's disease.

Medication	Amount mean(SD) mg	Number of patients
Levodopa	551.3 (239.8)	40
Ropinirole hydrochloride	22.6 (17.4)	19
Istradefylline	30.0 (10.4)	12
Entacapone	486.4 (317.9)	11
Rotigotine	14.9 (5.6)	10
Zonisamide	69.4 (89.9)	9
Amantadine hydrochloride	144.4 (76.8)	9
Opicapone	25.0 (0)	9
Trihexyphenidyl hydrochloride	3.6 (2.3)	7
Selegiline hydrochloride	4.6 (1.7)	7
Rasagiline mesylate	0.86 (0.24)	7
Safinamide mesylate	64.3 (24.4)	7
Pramipexole hydrochloride hydrate	2.5 (0.9)	3
Pergolide mesylate	0.5	1

This study was conducted in accordance with the principles embodied in the Declaration of Helsinki and was approved by the Institutional Review Board of Fukuoka Mirai Hospital (ethical approval number: 202206–1; June 1, 2022). Written informed consent was obtained from all patients. This manuscript adheres to the Recommendations for the Conduct, Reporting, Editing, and Publication of Scholarly Work in Medical Journals.

### 2.2 CPM recording paradigm

The CPM paradigm was employed according to Rossi et al.'s method [28], which combines a painful electrical stimulus (test stimulus) with a cold pressure task (conditioned stimulus). The CSP was recorded during the test stimulus with and without cold pressure.

#### 2.2.1 CSP

The CSP method was similar to that in our previous report [31]. The CSP during isometric contraction of the thumb while delivering cutaneous electrical stimuli to the index finger was recorded. Ring electrodes were placed over the D2 interphalangeal joints to apply constant square-wave electrical stimuli (0.5 ms), with the cathode and anode placed in the proximal and distal positions, respectively. The sensory threshold of electrical stimulation was determined, and stimulation of 15 times the sensory threshold (high-intensity stimulation) was delivered. Voluntary electromyographic activity was recorded using surface Ag/AgCl electrodes from the abductor pollicis brevis with reference to the thumb interphalangeal joint. The sensitivity was set at 200 μV/div, with a 10–5000 Hz bandpass filter. Electrical stimuli were delivered manually at an interstimulus interval of approximately 2–3 s. The patients were requested to maintain approximately 50 % of the maximum voluntary isometric contraction of the abductor pollicis brevis. Full-wave rectification was performed, and the electromyographic activity of ten trials was averaged. The analysis time consisted of pre- and post-stimuli of 90 and 360 ms, respectively.

#### 2.2.2 Test stimulus

Electrical stimulation of the right index finger was performed at a strength of 15 times the sensory threshold to record the CSP as the test stimulus. The patients were instructed to verbally rate the strength of electrical pain during the examination of CSP elicited by high-intensity stimulation of the right index finger on an NRS from 0 (“no pain”) to 10 (“most intense pain imaginable”).

#### 2.2.3 Conditioned stimulus

A cold pressure task was used as the conditioned stimulus. After recording the test stimulus-CSP and evaluating the NRS scores, the patients were instructed to dip their left hand up to the wrist into a bath with ice water (3–4 °C) and to verbally report the occurrence of cold pain sensation. When the patients reported cold pain sensation with NRS scores of more than 5/10, usually around 10 s after immersion (Table 3), we started to record the CSP with the same electrical strength just before the conditioned stimulus task. The cold pressure task ended after the CSP recording, and the average total immersion time in ice water was within 1 min on average (Table 3). Immediately after removing the left hand from the ice water, the patients were asked to verbally rate the pain in the right index finger on an NRS during the conditioned stimulus.

**Table 3:** Comparisons of experimental parameters, Hoehn and Yahr, and UPDRS part III during off and on states of anti-Parkinson's drugs.

	Drug off (*n* = 40)	Drug on (n = 40)	Paired t-test
Body temperature (Celsius)	36.3 (0.2)	36.2 (0.4)	ns
Left hand temperature (Before cold pressure)	31.0 (1.7)	31.4 (1.1)	ns

Immersion time			
Time to 5/10 NRS	8.8 (4.6)	10.3 (5.1)	ns
Total time	45.6 (11.2)	47.2 (9.5)	ns
Hoehn & Yahr	3.2 (0.8)	2.6 (0.6)	***p* < 0.01**
UPDRS part III	43.6 (15.5)	21.8 (14.1)	***p* < 0.05**

Data are given as mean (standard deviation).

NRS, numerous rating scale; UPDRS-III, Unified Parkinson's disease Rating Scale Part III；ns, no significance. Significant effects are shown in bold.

#### 2.2.4 Waveform analysis of the CSP

**Fig. 2:**
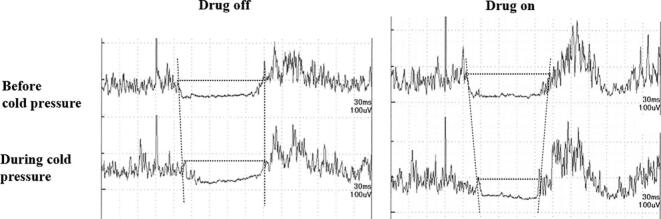
Representative changes in the cutaneous silent period (CSP) due to cold pressure in a 64-year-old female patient with Parkinson's disease (PD) from Group 1. Latency measurements were feasible because of minimal electromyographic contamination in the CSP. The CSP duration decreased during cold pressure, with a more pronounced change observed when the anti-PD medications was in the “on” state.

**Fig. 3:**
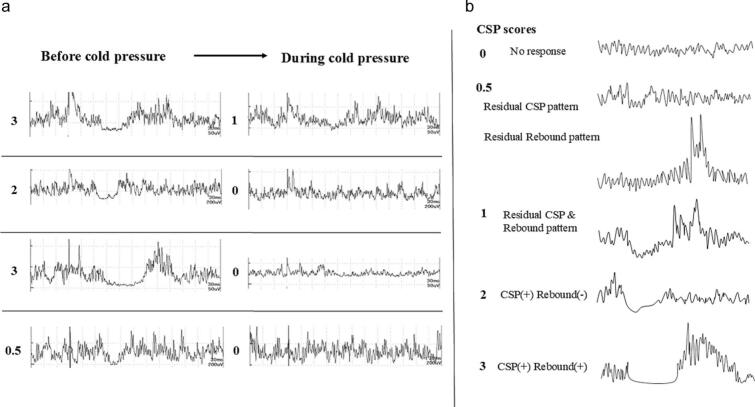
(a) Examples from four cases in Group 2, illustrating the difficulty in determining CSP latency during cold pressure. (b) Schematic representation of CSP scores ranging from “0” to “3.”

For the CSP analysis, the patients were divided into two groups. Group 1 (22 patients) comprised patients whose CSP waveforms were analyzed using latency measures because there was little electromyographic contamination in the silent period (Fig. 2), and Group 2 (18 patients) consisted of patients whose precise latency determination of CSP was disturbed by electromyographic contamination or in whom the waveform disappeared during the cold pressure test (Fig. 3a). In Group 2, the CSP score, which has been previously reported, was adopted (Fig. 3b). Depending on the waveform characteristics, one of the two measurement methods was selected.

#### 2.2.5 Latency analysis of CSP (duration)

The CSP latency analysis method in Group 1 was the same as that previously reported [31]. The baseline level was defined as 80 % of the amplitude between the trough and peak of the pre-stimulus electromyography in the rectified trace. A drop in the electromyographic trace below the baseline indicated the onset of CSP, and the end of CSP was defined as the point at which the trace returned to the baseline level. The duration of CSP was defined as latencies measured from the onset to the end of CSP. The presence of an electromyographic rebound was considered when its amplitude increased above the maximum peak of the pre-stimulus electromyography.

The duration of CSP was compared between the “on” and “off” states of anti-PD medications in Group 1.

#### 2.2.6 CSP score analysis

As reported elsewhere [31], the CSP scores ranged from 0 to 3 points (Fig. 3b). A score of 0 indicated no response. A score of 0.5 points was assigned for (1) a residual CSP pattern exhibiting a drop in the electromyographic trace below the baseline with contaminated electromyography without a subsequent electromyographic rebound or (2) a residual clear electromyographic rebound pattern without a CSP. A score of 1 point was given for a waveform displaying the association of residual CSP with electromyographic contamination and an electromyographic rebound pattern. A score of 2 points indicated a clear CSP with little or no electromyographic contamination. However, no electromyographic rebound was observed. A score of 3 points was assigned for a clear CSP with little electromyographic contamination and a clear electromyographic rebound. These scores were compared between the “on” and “off” states of anti-PD medications in Group 2.

### 2.3 Statistical analysis

For statistical analysis, BellCurve for Excel (version 4.08, Social Survey Research Information Co., Ltd., Japan) was used. A MANOVA was applied to assess the effect of the factor ***pain*** (patients with PD with and without clinical pain) on the patient's characteristics, including age, disease duration, MMSE, LEDD and UPDRS part III (motor score). A MANOVA with the same design and the factor ***pain*** was applied to investigate the effect of pain on the change of CSP duration (group 1) or CSP score (group 2) and the change of NRS. Paired *t*-tests were conducted to compare experimental parameters between the off and on states of anti-PD drugs. A two-way repeated measures ANOVA with the within-subject factor ***cold-c*** (CSP duration or score before and during cold pressure) and the between-subject factor ***drug*** (anti-PD drug off and on states) was performed to determine whether ***cold-c*** had a significant effect on the CSP duration or score and whether there was an interaction with the *drug* in group 1 and 2, respectively. Similarly, a two-way repeated measures ANOVA with the within-subject factor ***cold-n*** (NRS before and during cold pressure) and the between-subject factor ***drug*** was performed to determine whether ***cold-n*** had a significant effect on the NRS and whether there was an interaction with the ***drug*** in each group 1 and 2, respectively. Correlations between the CSP parameters and NRS scores were analyzed using Spearman's rank-order test. Similarly, correlations between the LEED and the changes in CSP parameters or NRS were examined. Statistical significance was set at α= 0.05. This study was conducted in collaboration with a statistical expert.

## 3 Results

### 3.1 Clinical parameters

A MANOVA with group factor ***pain*** and the age, the disease duration, the MMSE, the LEDD, and the UPDRS part III at drug on the state as dependent variables did not show a significant main effect of ***pain*** [F(5,34)=0.37; *p*=0.865] on these variables. Comparisons of these dependent variables between PDN and PDP showed no significant difference (Table 1).

### 3.2 Experimental parameters

There was no significant difference in body and left-hand temperatures before cold pressure and time to reach pain (5/10 NRS) during cold pressure and total immersion time between the off and on of anti-PD drugs (Table 3). Hoehn and Yahr stage and UPDRS part III scores were significantly decreased during drug-on-state when compared with those during drug-off-state (Table 3).

### 3.3 Effects of clinical pain and anti-PD drugs (on/off) on CSP and NRS

The effects of anti-Parkinson drugs (both on and off conditions) and clinical pain on the changes of CSP duration (or CSP score) and NRS were evaluated in group 1 (Table 4) and group 2 (Table 5). A MANOVA with factor ***pain*** and the changes of CSP duration and the change of NRS as dependent variables did not show a significant influence of pain in group 1 [F(4,17)=1.15; *p*=0.367]. Similarly, comparisons of these dependent variables between PDN and PDP showed no significant difference regardless of the drug on or off conditions (Table 4).

**Table 4:** Effect of anti-Parkinson drugs on changes of cutaneous silent period duration and numerous rating scale in group 1; descriptive statistics and MANOVA results.

Group 1 (*n* = 22)	PDN (*n* = 5)	PDP (*n* = 17)	PDN vs PDP (p value)
Drug off			
Changes of CSP duration	14.6 (11.2)	10.2 (7.0))	0.29
Changes of NRS	0.7 (1.4)	0.3 (1.9)	0.66

Drug on			
Changes of CSP duration	30.7 (12.5)	19.5 (10.9)	0.06
Changes of NRS	1.4 (1.5)	1.1 (1.5)	0.72

Changes of CSP duration and NRS = (values before cold pressure) − (values during cold pressure).

Data are given as mean (standard deviation).

CSP, cutaneous silent period; NRS, numerous rating scale; PDN, Parkinson's disease without pain; PDP, Parkinson's disease with pain.

**Table 5:** Effect of anti-Parkinson drugs on changes of cutaneous silent period score and numerous rating scale in group 2; descriptive statistics and MANOVA results.

Group 2 (*n* = 18)	PDN (*n* = 6)	PDP (*n* = 12)	PDN vs PDP (*p* value)
Drug off			
Changes of CSP score	1.2 (1.2)	0.3 (0.9)	0.08
Changes of NRS	0.8 (0.4)	0.6 (0.8)	0.15

Drug on			
Changes of CSP score	0.5 (0.4)	1.2 (1.0)	0.48
Changes of NRS	0.7 (0.8)	0.7(1.2)	1.00

Changes of CSP score and NRS = (values before cold pressure) − (values during cold pressure).

Data are given as mean (standard deviation).

CSP, cutaneous silent period; NRS, numerous rating scale; PDN, Parkinson's disease without pain; PDP, Parkinson's disease with pain.

A MANOVA with factor ***pain*** and the changes of CSP score and the change of NRS as dependent variables did not show a significant influence of pain in group 2 [F(4,13)=1.15; *p*=0.377]. Likewise, comparisons of these dependent variables between PDN and PDP showed no significant difference, irrespective of the drug condition (Table 5).”

### 3.4 Effects of cold pressure and anti-PD drugs on CSP and NRS

#### 3.4.1 Group 1

Table 6 shows the effects of cold pressure and anti-PD drugs on CSP duration and NRS in group 1. Regarding the CSP duration in group 1, a two-way repeated measures ANOVA revealed a significant main effect of ***cold-c*** [F(1,42)=117.15; *p*<0.001], no significant effect of ***drug*** [F(1,42)=0.003; *p*=0.961] but a significant ***cold-c*** **×** ***drug*** interaction [F(1,42)=12.38; *p*=0.001]. This indicates that the effect of cold pressure on CSP duration differed depending on the presence or absence of anti-PD drugs. Therefore, we examined the change in CSP duration due to cold pressure under both drug-on and drug-off conditions. The change in CSP duration was calculated as: (CSP duration before cold pressure) – (CSP duration during cold pressure). This change was significantly greater in the drug on state [22.1(12.0) msec] compared to the drug off state [11.2(8.0) msec] (*p*=0.0003). Regarding the NRS in group 1, there was a significant main effect of ***cold-n*** [F(1,42)=10.21; *p*=0.003] but no significant effect of ***drug*** [F(1,42)=0.11; *p*=0.748] and no significant ***cold-n*×*drug*** interaction [F(1,42)=2.63; *p*=0.113]. Conversely, the change in NRS score was significantly correlated with the change in CSP duration (rs=0.45, *p*=0.034, Table 7) when the patient was drug-on-state.

**Table 6:** Effects of cold pressure and anti-Parkinson drugs on cutaneous silent period (CSP) duration and numerous rating scale in group 1 (*n* = 22).

Group 1 (n = 22)	Before cold pressure	During cold pressure
CSP duration (msec)		
Drug on	102.9 (37.3)	80.9 (30.2)
Drug off	97.0 (32.8)	85.8 (29.8)

Numerous rating scale		
Drug on	8.1 (1.8)	7.0 (1.9)
Drug off	7.6 (1.9)	7.2(2.1)

Data are given as mean (standard deviation).

CSP, cutaneous silent period.

**Table 7:** Correlation between the change of NRS and that of CSP duration in group 1 (n = 22).

NRS change⁎	CSP duration change⁎	Statistics (Spearman)	Correlation coefficient
1.2 (1.5)	22.1 (12.0)	**p = 0.034**	**0.45**

NRS, numerous rating scale; CSP, cutaneous silent period.

Data are given as mean (standard deviation); Significant effects are shown in bold.

⁎ Changes of NRS and CSP duration = (values before cold pressure) − (values during cold pressure).

#### 3.4.2 Group 2

Table 8 shows the effect of cold pressure and the anti-PD drugs on CSP score and NRS in group 2. Regarding the CSP score in group 2, there was a significant main effect of ***cold-c*** [F(1,34)=18.85; *p*<0.001] but no significant effect of ***drug*** [F(1,34)=1.89; *p*=0.178] and no significant ***cold-c*×*drug*** interaction [F(1,34)=1.51; *p*=0.228]. Regarding the NRS in group 2, there was a significant main effect of ***cold-n*** [F(1,34)=20.92; *p*<0.001] but no significant effect of ***drug*** [F(1,34)=0.18; *p*=0.677] and no significant ***cold-n*×*drug*** interaction [F(1,34)<0.01; *p*>0.99]. In Group 2, there was no significant correlation between changes in the NRS and CSP scores in spite of the drug-on-state (Table 9).

**Table 8:** Effects of cold pressure and anti-Parkinson drugs on cutaneous silent period (CSP) score and numerous rating scale in group 2 (*n* = 18).

	Before cold pressure	During cold pressure
CSP score		
Drug on	2.6(0.8)	1.7(1.0)
Drug off	2.0(1.0)	1.5(1.2)

Numerous rating scale		
Drug on	7.6(1.6)	6.9(1.8)
Drug off	7.3(1.5)	6.7(1.7)

CSP, cutaneous silent period. Data are given as mean(standard deviation).

**Table 9:** Correlation between the change of NRS and that of CSP score in group 2 (n = 18).

NRS change⁎	CSP score change⁎	Statistics (Spearman)
0.7 (1.0)	0.9 (0.9)	ns

NRS, numerous rating scale; CSP, cutaneous silent period; ns, no significance.

Data are given as mean (standard deviation).

⁎ Changes of NRS and CSP score = (values before cold pressure) − (values during cold pressure).

### 3.5 The effect of LEDD on CPM parameters

There was no statistically significant correlation between LEDD and the changes in CSP duration in group 1 (rs =0.40, *p*=0.066), CSP score in group 2 (rs =0.44, *p*= 0.071), NRS in group 1 (rs=0.10, *p*=0.673), or NRS in group 2 (rs =0.26, *p*=0.299).

## 4 Discussion

Building on our hypothesis that anti-Parkinson's drugs may enhance CPM in patients with PD, this study evaluated both subjective (NRS) and objective (CSP) measures to assess such effects.

### 4.1 Main findings

The main findings were as follows:

1. Both patients with PD with and without clinical pain showed reductions in NRS scores, CSP duration, and CSP scores during the CPM test, but there was no significant difference in the magnitude of CPM, whether the patient suffered from pain or not (Table 4, Table 5). These findings suggest that CPM dysfunction is not the primary contributor to clinical pain in PD.
2. Anti-PD drugs alone did not significantly affect CSP duration. However, during the cold pressor test, the drugs had a notable effect, and the change in CSP duration was significantly greater when patients were on medication compared to when they were off (Table 6). These interesting findings suggest that dopaminergic drugs may enhance CPM capacity. It might further be hypothesized that dopaminergic interventions may influence ‘reactive’ CPM control in response to external stress (cold) rather than sustained analgesia.
3. Changes in CSP duration were significantly correlated with changes in NRS scores (Table 7), indicating a link between objective and subjective measures of pain modulation.
4. Although CSP score analysis effectively detected CPM, it did not show a significant correlation with changes in NRS scores (Table 8, Table 9). This result indicates that a CSP score method was not sensitive enough to reveal a link between objective and subjective measures of pain modulation. The CSP score method is used exclusively in cases of high signal noise, and it might not precisely reflect the CSP change when compared to the CSP duration measurement. Therefore, its correlation with subjective assessment would tend to be weaker.
5. LEDDs were not correlated with the changes in CSP or NRS, suggesting that the dosage of dopaminergic drugs might not be directly related to the CPM. There seem to be complex factors that cannot be captured by LEDDs alone, such as the influence of other neurotransmitters (e.g. noradrenaline and serotonin) or individual sensitivities to induce the changes in CSP and NRS.

### 4.2 “Pain inhibits pain” and CSP studies

Contemporary research has explored the “pain inhibits pain” phenomenon [32,33] through the mechanisms of DNIC and CPM. Although the influence of DNIC from cortical and subcortical regions has gradually been clarified [[33], [34], [35]], a spino-bulbo-spinal loop, including SRD, has been suggested as the primary mechanism of DNIC and CPM by affecting WDR neurons across the whole spinal cord [14,36,37]. Alongside this loop, classical descending inhibitory pathways, including those in the PAG and rostral ventromedial medulla (RVM), have also been implicated in DNIC/CPM [33,38,39].

Initially, DNIC/CPM activation was thought to inhibit WDR neuron activity primarily via SDR in the caudal medulla [36,37,39,40]. However, accumulated evidence suggests a pronociceptive function whereby the DNIC/CPM loop fundamentally enhances WDR neuron activity and wind-up [36,[40], [41], [42]]. This mechanism may facilitate withdrawal from the source of pain during tissue injury, a process linked to reflexes such as the nociceptive flexion reflex and CSP, which is used in this study. During CPM activation, this pronociceptive response appears to be modulated, possibly suppressing excessive pain to support the healing process. Recent human studies have hypothesized that SRD may tonically inhibit CPM, with its deactivation activating the descending noradrenergic pathway from the locus coeruleus, thereby exerting spinal pain control [17,[43], [44], [45]]. Impairments in this function may be related to chronic pain development, suggesting that CPM effects vary across injury stages [44].

In recent CPM evaluations, Rossi et al. proposed a novel objective approach using the CSP in cold pressure tests [28]. Their findings, consistent with ours, showed decreased CSP duration under cold pressure (Fig. 2; Table 6). This study expands on this by examining changes in CSP score (waveform patterns), which may serve as additional markers for CPM evaluation (Fig. 3a, b; Table 8). Shortened CSP duration and diminished CSP score are common when the stimulation intensity is insufficient to elicit clear CSP waveforms [28,31,46], suggesting that our observed results during cold pressure reflect a decrease in pain signals, likely due to CPM activation.

CSP duration represents 2 main pathways including the segmental intrinsic Aδ pain pathway and possible spino-bulbo (and/or cortical)-spinal Aβ pathway [28,46]. It is known that the duration of the former is longer than that of the latter [28,31,46]. CSP elicited by an Aβ fiber stimulation does not show latency changes during CPM using cold pressure [28]. Thus, CSP shortening due to CPM implementation represents suppression of the Aδ pain pathway.

According to the hypothesis proposed by Rossi [28], test stimulus of an Aδ fiber may activate the WDR neuron, followed by activation of the inhibitory interneuron, resulting in decreased α-motoneuron activity. This process produces CSP. When a conditioned stimulus is applied, the WDR neuron is suppressed by the descending inhibitory fiber, and deactivation of the inhibitory interneuron increasesα-motoneuron activity. During this CPM, CSP duration is shortened and/or clear CSP waveform formation is disturbed.

### 4.3 Clinical pain in patients with PD

In the present study, there were no significant differences in CPM as assessed by changes in objective CSP and subjective NRS between PDP and PDN. These findings indicate that CPM dysfunction may not directly explain the presence of clinical pain in PD, suggesting that other mechanisms—possibly peripheral or affective components—may contribute more significantly [47]. This aligns with previous reports that intermittent PD-related pain may exert less influence on the CPM pathway [3].

### 4.4 Effects of anti-PD drugs on CSP and CPM

This study demonstrated that anti-PD drugs did not significantly affect the duration of CSP. This finding is consistent with our previous results [28], as well as those reported by Nakashima et al. [48], which suggest that anti-PD drugs exert minimal influence on CSP latency. This is likely because CSP latency, when elicited by strong electrical stimulation, remains relatively stable regardless of pharmacological intervention [31].

However, this present study also revealed that the effect of anti-PD medication became pronounced during the cold pressor test, suggesting dopaminergic drugs enhance CPM capacity. This contrasts with prior studies reporting no significant CPM changes in patients with PD [3,20,21]. In these studies, the degree of CPM in patients with PD—evaluated during temporary withdrawal from anti-PD medication—did not significantly differ from that of healthy controls [3,20]. Granshorn et al. [21] specifically tested CPM in drug-naïve patients with de novo PD to eliminate the effects of long-lasting anti-PD drugs, also showing no difference in CPM response from healthy controls. Furthermore, anti-PD medication was not found to influence CPM responses in these patients [20], although they noted a non-significant trend toward drug-related CPM changes [20].

A key difference between our study and previous studies may lie in the clinical profiles of the participants. PD severity in those studies was relatively mild; for instance, one study included 16 patients of Hoehn & Yahr stage I or II out of 17 drug-naïve de novo patients with PD [21], and another study included no patients with PD beyond stage II [20]. In contrast, our cohort consisted of 40 patients hospitalized for stereotactic surgery, of whom 21 were classified as stage III or above even during the “on” medication state, indicating a more advanced disease stage. These differences might explain the discrepancies in results, as CSP and CPM modulation by anti-PD drugs may differ significantly between early-stage patients with PD—who typically respond well to L-DOPA—and those in advanced stages, where drug efficacy is reduced or fluctuating. Good response to L-DOPA would enhance the pain suppression system as a whole, but might mask the function of CPM in some circumstances. To clarify this issue, further research is needed to characterize the effects of anti-PD drugs on CPM across various conditions or stages of PD.

Besides participant characteristics, methodological differences in study design may also account for divergent findings. Our study employed CSP analysis using an electrical test stimulus combined with a cold pressor test as the conditioning stimulus. In contrast, Mylius et al. used an electrical test stimulus with a heat conditioning stimulus [3], while Grashorn et al. studies utilized a heat test stimulus in combination with a cold pressor conditioning stimulus [20,21]. These variations in the type and pairing of stimuli might influence the observed CPM responses [15].

Conversely, these findings correspond with several clinical observations in which dopaminergic treatments have been shown to significantly elevate pain thresholds in patients with PD [4,8,49,50], though some found no differences between drug-on and drug-off periods [5]. These results collectively underscore the critical role of dopaminergic pathways in the modulation of nociception. Complementary evidence comes from Treister et al., who demonstrated enhanced CPM in healthy individuals following the administration of the dopamine agonist apomorphine [51]. Additionally, PET studies have shown that L-DOPA increases cold pain thresholds in patients with PD while reducing activity in brain regions associated with sensory-discriminative, cognitive, and affective-motivational components of pain processing [6]. Another PET study demonstrated a relationship between striatal dopamine release and ice water immersion-induced pain inhibition, implicating striatal dopamine relation in CPM in healthy participants [25]. Nevertheless, earlier research involving patients with PD failed to detect significant effects of anti-PD medication on CPM [20]. Against this backdrop, our study is the first to demonstrate a significant enhancement of CPM by anti-PD medications in patients with PD, as assessed via CSP modulation. This novel finding proposes the potential utility of CSP-based methodologies in detecting pharmacological influences on endogenous pain inhibitory mechanisms in PD.

### 4.5 CPM evaluated by the NRS and CSP

This study revealed that the cold pressure test induced a significant decrease in the NRS scores in the CSP duration analysis of Group 1 (Table 6) and in the CSP score analysis of Group 2 (Table 8), in agreement with the results of previous studies on cold pressure on the CPM analyzed by the NRS [52,53].

The NRS scores were significantly correlated with the duration of CSP latency; however, the correlation coefficient (0.45) was not so high but moderate (Table 7). The CSP score showed no significant correlation with the NRS scores (Table 9). These results suggest that the pain-processing layer differs between the CSP and NRS. This difference may be attributed to the fundamental neurophysiological nature of CSP at the spino-bulbo-spinal levels [31], whereas the NRS reflects a more complex psychological-cognitive process [15].

### 4.6 Role of the dopaminergic system in DNIC/CPM

This study indicates a potential modulating function of the dopaminergic system in pain perception through the DNIC/CPM, although the role of dopamine in the DNIC has traditionally been considered minor [45,54]. Due to the heterogeneity of anti-Parkinsonian drug combinations in this study (Table 2), it was not feasible to isolate the effects of individual medications. Therefore, we instead examined whether the overall LEDD might influence CPM capacity, as reflected by changes in the CSP parameters and NRS scores. However, no significant correlations were observed between LEDD and alterations in either CSP or NRS. These findings would be due to the mechanisms underlying CPM; they are not solely dopaminergic but are likely to involve additional neurotransmitter systems, such as the serotonergic, noradrenergic, or opioidergic pathways.

Anatomically, the integrity of the ipsilateral dorsolateral funiculus and medullary SRD is essential for the DNIC/CPM mechanism [17,43,45,55,56]. SRD lesions impair the DNIC, whereas lesions in the PAG or RVM, associated with opioidergic and serotonergic pathways, do not affect the DNIC [45,57,58]. Moreover, SRD has extensive anatomical connections to the medulla, cerebellum, pons, mesencephalon, hypothalamus, thalamus, and telencephalon, including the globus pallidus [59], suggesting its central role in DNIC/CPM modulation.

Pharmacologically, noradrenaline, serotonin, and endogenous opioids are all crucial for DNIC/CPM function maintenance [17,45,57,60]. Some opinions claim that dopamine's descending pathways may not directly mediate spinal “pain inhibits pain” effects [60], as the expression of dopamine receptors in the spinal cord appears to be scarce [60,61]. Buhidma et al. have shown that unilateral nigral-dopaminergic depletion causes a reduction in dopaminergic tone in the PAG, and that leads to reduced activity in the serotonergic neurons in the RVM, thereby reducing opioidergic tone in the dorsal horn of the spinal cord [18]. Unlike dopamine agonists, L-DOPA does not specifically increase dopamine signalling in the brain but also increases noradrenergic signalling. Therefore, the impact of noradrenergic signalling on CPM in PD has to be included [17,45,60]. Given that L-DOPA is metabolized to dopamine and noradrenaline, it may also exhibit pseudo-serotonergic effects, which account for some analgesic effects [62,63]. Therefore, it remains uncertain whether dopamine acts directly on pain or via other neurotransmitters, such as noradrenaline and serotonin. Conversely, fiber connections between the nucleus accumbens and spinal cord directly, or interposed by the amygdala, have been clarified [64], and an experimental study showed that the antinociceptive effect via DNIC was blocked by dopamine antagonist injection into the nucleus accumbens [65]. Descending dopaminergic projections from the hypothalamic nucleus can also inhibit pain [66,67] by activating D2 receptors in the dorsal horn [67]. WDR neuron windup and DNIC are inhibited by activating and blocking, respectively these D2 receptors [67]. Antinociceptive and pronociceptive actions could also be mediated by D2 and D1, respectively, in the spinal dorsal horn [68], suggesting that a D2 agonist or a dopamine precursor (L-DOPA) may provide pain relief [68].

### 4.7 Limitations

Patients with PD were divided into two categories with or without clinical pain along with UPDRS part II, but more detailed pain classification using the King's Parkinson's disease pain scale [69] was not utilized in this study, so the detailed analysis in accordance to the pain type could not be completed. However, it was at least possible to show that there was no CPM difference between PDP and PDN, similar to the previous study results [3] using Ford's classification of PD pain [70].

We used 15 sensory threshold electrical stimulation intensity to record the CSP, which primarily activates Aβ and Aδ fibers [31,46,71], whereas C-fiber conduction, which was too slow to contribute to the time window of electrically elicited CSP [71], was not involved. Therefore, comparing DNIC findings from animal studies that activate C fibers [14,37] with human CPM results, such as the present one, requires caution.

Most participants were on various anti-PD medications, and the 20-h off-medication period was too short to fully eliminate the effects of dopamine receptor agonists [31]. This significantly interfered with this study. However, even with interference factors, the symptoms “on time” and “off time” of PD patients are significant (Table 3), and there is still an impact of dopamine fluctuations on pain detection (Table 6, Table 7). Therefore, the results of this study can potentially establish the relationship between dopamine and pain detection.

Another important limitation is the lack of a control group to verify whether our finding was specific to PD patients in general or PD patients being treated specifically with carbidopa and levodopa. Could this be an isolated effect present with the use of dopaminergic medication in any condition? Or is this an effect that is lost in patients with PD only when compared to controls and is it ameliorated by dopaminergic medications? These are questions that can only be answered with a control group.

It should also be noted that all participants in this study were patients with advanced PD who had been admitted for surgical procedures. As such, the findings may not be generalizable to individuals in earlier stages of the disease. This selection bias may have influenced the observed enhancement of descending pain inhibitory mechanisms under antiparkinsonian treatment. Future studies involving a broader spectrum of disease severity will be essential to validate the generalizability of these findings across various stages of the PD population.

## 5 Conclusion

This study demonstrated that CPM can be assessed using the objective electrophysiological method of CSP. The results of patients with PD align with the 10 %–60 % range of neurophysiological changes of CPM observed in healthy subjects [22]. These findings support the potential use of CSP as a neurophysiological biomarker for assessing CPM in PD, which may aid in future therapeutic monitoring and personalized pain management strategies. This represents the first study to demonstrate that anti-PD medications enhance CPM in patients with PD. Further investigation is required to clarify the specific involvement of dopamine in DNIC/CPM.

## Data collection methods

Data was collected through patient medical records, computer analysis of electromyographic waveforms, and information obtained from patients during experiments.

## Ethical considerations including consent to participate and consent for publication

This study was approved by the Ethics Committee of Fukuoka Mirai Hospital (Ethics Code: 202206–1) on June 1, 2022. All participants provided written informed consent prior to enrolment in the study. This research was conducted ethically in accordance with the World Medical Association Declaration of Helsinki. This manuscript adheres to the Recommendations for the Conduct, Reporting, Editing, and Publication of Scholarly Work in Medical Journals.

## Author contribution

The content of this manuscript is accurate and represents the authors' work and opinions, not those of the sponsoring agent. The corresponding author agrees to communicate with all other authors and will obtain their approval for the final version to be published. All authors have made substantial contributions to the research design and acquisition, analysis, or interpretation of data; actively participated in drafting and critically revising the manuscript; and provided final approval of the submitted version.

## CRediT authorship contribution statement

**Eiichirou Urasaki:** Writing – original draft, Methodology, Investigation, Formal analysis, Data curation, Conceptualization. **Yasushi Miyagi:** Writing – review & editing, Supervision, Data curation. **Junji Kishimoto:** Writing – review & editing, Methodology, Formal analysis.

## Funding statement

This study did not receive any specific grants from funding agencies in the public, commercial, or nonprofit sectors.

## Declaration of competing interest

Eiichirou Urasaki, Yasushi Miyagi, and Junji Kishimoto have no conflicts of interest to declare.

## Data Availability

Primary and supplementary data are openly available at the figshare depository. DOI https://doi.org/10.6084/m9.figshare.28137296.

## Appendix A Supplementary data

Supplementary material 1 EU Data-1: Age, sex, and medications used in patients with Parkinson's disease. Supplementary material 1

Supplementary material 2 EU Data-2: Experimental parameters during off and on states of anti-Parkinson drugs. Supplementary material 2

Supplementary material 3 EU Data-3: Changes in cutaneous silent period latencies in group 1 (Drug on/off, Cold pressure on/off states). Supplementary material 3

Supplementary material 4 EU Data-4: Changes in NRS in group 1 (Drug on/off, Cold pressure on/off states). Supplementary material 4

Supplementary material 5 EU Data-5: Changes in cutaneous silent period scores in group 2 (Drug on/off, Cold pressure on/off states). Supplementary material 5

Supplementary material 6 EU Data-6: Changes in NRS in group 2 (Drug on/off, Cold pressure on/off states). Supplementary material 6

## References

[1] Nègre-Pagès L., Regragui W., Bouhassira D., Grandjean H., Rascol O., DoPaMiP Study Group (2008 Jul 30). Chronic pain in Parkinson’s disease: the cross-sectional French DoPaMiP survey. Mov. Disord..

[2] Viseux F.J.F., Delval A., Simoneau M., Defebvre L. (2023 May). Pain and Parkinson’s disease: current mechanism and management updates. Eur. J. Pain.

[3] Mylius V., Engau I., Teepker M., Stiasny-Kolster K., Schepelmann K., Oertel W.H., Lautenbacher S., Möller J.C. (2009 Jan). Pain sensitivity and descending inhibition of pain in Parkinson’s disease. J. Neurol. Neurosurg. Psychiatry.

[4] Battista A.F., Wolff B.B. (1973 Jul). Levodopa and induced-pain response. A study of patients with Parkinsonian and pain syndromes. Arch. Intern. Med..

[5] Djaldetti R., Shifrin A., Rogowski Z., Sprecher E., Melamed E., Yarnitsky D. (2004 Jun 22). Quantitative measurement of pain sensation in patients with Parkinson disease. Neurology.

[6] Brefel-Courbon C., Payoux P., Thalamas C., Ory F., Quelven I., Chollet F., Montastruc J.L., Rascol O. (2005 Dec). Effect of levodopa on pain threshold in Parkinson’s disease: a clinical and positron emission tomography study. Mov. Disord..

[7] Granovsky Y., Schlesinger I., Fadel S., Erikh I., Sprecher E., Yarnitsky D. (2013 Oct). Asymmetric pain processing in Parkinson’s disease. Eur. J. Neurol..

[8] Gerdelat-Mas A., Simonetta-Moreau M., Thalamas C., Ory-Magne F., Slaoui T., Rascol O., Brefel-Courbon C. (2007 Oct). Levodopa raises objective pain threshold in Parkinson’s disease: a RIII reflex study. J. Neurol. Neurosurg. Psychiatry.

[9] Perrotta A., Sandrini G., Serrao M., Buscone S., Tassorelli C., Tinazzi M., Zangaglia R., Pacchetti C., Bartolo M., Pierelli F., Martignoni E. (2011 Feb 15). Facilitated temporal summation of pain at spinal level in Parkinson’s disease. Mov. Disord..

[10] Guieu R., Pouget J., Serratrice G. (1992). Seuil nociceptif et maladie de Parkinson [Nociceptive threshold and Parkinson disease]. Rev. Neurol. (Paris).

[11] Li J., Ji Y.P., Qiao J.T., Dafny N. (1992 Sep 18). Suppression of nociceptive responses in parafascicular neurons by stimulation of substantia nigra: an analysis of related inhibitory pathways. Brain Res..

[12] Chudler E.H., Dong W.K. (1995 Jan). The role of the basal ganglia in nociception and pain. Pain.

[13] Heinricher M.M., Tavares I., Leith J.L., Lumb B.M. (2009 Apr). Descending control of nociception: specificity, recruitment and plasticity. Brain Res. Rev..

[14] Le Bars D., Dickenson A.H., Besson J.M. (1979 Jun). Diffuse noxious inhibitory controls (DNIC). I. Effects on dorsal horn convergent neurones in the rat. Pain.

[15] Ramaswamy S., Wodehouse T. (2021 Jun). Conditioned pain modulation-a comprehensive review. Neurophysiol. Clin..

[16] Domenici R.A., Campos A.C.P., Maciel S.T., Berzuino M.B., Hernandes M.S., Fonoff E.T., Pagano R.L. (2019 May). Parkinson’s disease and pain: modulation of nociceptive circuitry in a rat model of nigrostriatal lesion. Exp. Neurol..

[17] Sirucek L., Ganley R.P., Zeilhofer H.U., Schweinhardt P. (2023 Mar 1). Diffuse noxious inhibitory controls and conditioned pain modulation: a shared neurobiology within the descending pain inhibitory system?. Pain.

[18] Buhidma Y., Hobbs C., Malcangio M., Duty S. (2023 Apr 26). Periaqueductal grey and spinal cord pathology contribute to pain in Parkinson’s disease. NPJ Parkinsons Dis..

[19] Charles K.A., Naudet F., Bouali-Benazzouz R., Landry M., De Deurwaerdère P., Fossat P., Benazzouz A. (2018 Jul). Alteration of nociceptive integration in the spinal cord of a rat model of Parkinson’s disease. Mov. Disord..

[20] Grashorn W., Schunke O., Buhmann C., Forkmann K., Diedrich S., Wesemann K., Bingel U. (2015 Aug 13). Influence of dopaminergic medication on conditioned pain modulation in Parkinson’s disease patients. PLoS One.

[21] Grashorn W., Fründt O., Buhmann C., Wrobel N., Schmidt K., Bingel U. (2019 Aug 26). Conditioned pain modulation in drug-naïve patients with de novo Parkinson’s disease. Neurol. Res. Pract..

[22] Pud D., Granovsky Y., Yarnitsky D. (2009 Jul). The methodology of experimentally induced diffuse noxious inhibitory control (DNIC)-like effect in humans. Pain.

[23] Jurth C., Rehberg B., von Dincklage F. (2014). Reliability of subjective pain ratings and nociceptive flexion reflex responses as measures of conditioned pain modulation. Pain Res. Manag..

[24] Squintani G., Rasera A., Segatti A., Concon E., Bonetti B., Valeriani M., Tinazzi M. (2021 Mar). Conditioned pain modulation affects the N2/P2 complex but not the N1 wave: a pilot study with laser-evoked potentials. Eur. J. Pain.

[25] Hagelberg N., Martikainen I.K., Mansikka H., Hinkka S., Någren K., Hietala J., Scheinin H., Pertovaara A. (2002 Sep). Dopamine D2 receptor binding in the human brain is associated with the response to painful stimulation and pain modulatory capacity. Pain.

[26] Youssef A.M., Macefield V.G., Henderson L.A. (2016 Jul). Cortical influences on brainstem circuitry responsible for conditioned pain modulation in humans. Hum. Brain Mapp..

[27] Mills E.P., Keay K.A., Henderson L.A. (2021 Dec 21). Corrigendum: brainstem pain-modulation circuitry and its plasticity in neuropathic pain: insights from human brain imaging investigations. Front. Pain Res. (Lausanne).

[28] Rossi P., Pierelli F., Parisi L., Perrotta A., Bartolo M., Amabile G., Serrao M. (2003 Jan). Effect of painful heterotopic stimulation on the cutaneous silent period in the upper limbs. Clin. Neurophysiol..

[29] Postuma R.B., Berg D., Stern M., Poewe W., Olanow C.W., Oertel W., Obeso J., Marek K., Litvan I., Lang A.E., Halliday G., Goetz C.G., Gasser T., Dubois B., Chan P., Bloem B.R., Adler C.H., Deuschl G. (2015 Oct). MDS clinical diagnostic criteria for Parkinson’s disease. Mov. Disord..

[30] Tomlinson C.L., Stowe R., Patel S., Rick C., Gray R., Clarke C.E. (2010 Nov 15). Systematic review of levodopa dose equivalency reporting in Parkinson’s disease. Mov. Disord..

[31] Urasaki E., Miyagi Y., Kishimoto J. (2022 Aug). Effects of medications and subthalamic nucleus-deep brain stimulation on the cutaneous silent period in patients with Parkinson’s disease. Neuromodulation.

[32] Willer J.C., Bouhassira D., Le Bars D. (1999 Oct). Bases neurophysiologiques du phénomène de contre-irritation: les contrôles inhibiteurs diffus induits par stimulations nociceptives [Neurophysiological bases of the counterirritation phenomenon: diffuse control inhibitors induced by nociceptive stimulation]. Neurophysiol. Clin..

[33] Sprenger C., Bingel U., Büchel C. (2011 Feb). Treating pain with pain: supraspinal mechanisms of endogenous analgesia elicited by heterotopic noxious conditioning stimulation. Pain.

[34] Piché M., Arsenault M., Rainville P. (2009 Nov 11). Cerebral and cerebrospinal processes underlying counterirritation analgesia. J. Neurosci..

[35] Moont R., Crispel Y., Lev R., Pud D., Yarnitsky D. (2011 Jul). Temporal changes in cortical activation during conditioned pain modulation (CPM), a LORETA study. Pain.

[36] Villanueva L., Le Bars D. (1995). The activation of bulbo-spinal controls by peripheral nociceptive inputs: diffuse noxious inhibitory controls. Biol. Res..

[37] Le Bars D. (2002 Oct). The whole body receptive field of dorsal horn multireceptive neurones. Brain Res. Brain Res. Rev..

[38] Reynolds D.V. (1969 Apr 25). Surgery in the rat during electrical analgesia induced by focal brain stimulation. Science.

[39] Chebbi R., Boyer N., Monconduit L., Artola A., Luccarini P., Dallel R. (2014 Jun). The nucleus raphe magnus OFF-cells are involved in diffuse noxious inhibitory controls. Exp. Neurol..

[40] Lima D., Almeida A. (2002 Feb). The medullary dorsal reticular nucleus as a pronociceptive centre of the pain control system. Prog. Neurobiol..

[41] Almeida A., Størkson R., Lima D., Hole K., Tjølsen A. (1999 Jan). The medullary dorsal reticular nucleus facilitates pain behaviour induced by formalin in the rat. Eur. J. Neurosci..

[42] Dugast C., Almeida A., Lima D. (2003 Aug). The medullary dorsal reticular nucleus enhances the responsiveness of spinal nociceptive neurons to peripheral stimulation in the rat. Eur. J. Neurosci..

[43] Youssef A.M., Macefield V.G., Henderson L.A. (2016 Jan 1). Pain inhibits pain; human brainstem mechanisms. Neuroimage.

[44] de Melo P.S., Pacheco-Barrios K., Marduy A., Vasquez-Avila K., Simis M., Imamura M., Cardenas-Rojas A., Navarro-Flores A., Batistella L., Fregni F. (2024 Jul 7). The endogenous pain modulatory system as a healing mechanism: a proposal on how to measure and modulate it. Neuroscience.

[45] Bannister K., Dickenson A.H. (2017 Jul 1). The plasticity of descending controls in pain: translational probing. J. Physiol..

[46] Serrao M., Parisi L., Pierelli F., Rossi P. (2001 Nov). Cutaneous afferents mediating the cutaneous silent period in the upper limbs: evidences for a role of low-threshold sensory fibres. Clin. Neurophysiol..

[47] Tai Y.C., Lin C.H. (2019 Nov 28). An overview of pain in Parkinson’s disease. Clin. Park. Relat. Disord..

[48] Nakashima K., Takahashi K. (1992). Silent periods in the abductor pollicis brevis muscle in patients with Parkinson’s disease. Electromyogr. Clin. Neurophysiol..

[49] Schestatsky P., Kumru H., Valls-Solé J., Valldeoriola F., Marti M.J., Tolosa E., Chaves M.L. (2007 Dec 4). Neurophysiologic study of central pain in patients with Parkinson disease. Neurology.

[50] Brefel-Courbon C., Ory-Magne F., Thalamas C., Payoux P., Rascol O. (2013 May). Nociceptive brain activation in patients with neuropathic pain related to Parkinson’s disease. Parkinsonism Relat. Disord..

[51] Treister R., Pud D., Eisenberg E. (2013 Aug 26). The dopamine agonist apomorphine enhances conditioned pain modulation in healthy humans. Neurosci. Lett..

[52] Granot M., Weissman-Fogel I., Crispel Y., Pud D., Granovsky Y., Sprecher E., Yarnitsky D. (2008 May). Determinants of endogenous analgesia magnitude in a diffuse noxious inhibitory control (DNIC) paradigm: do conditioning stimulus painfulness, gender and personality variables matter?. Pain.

[53] Edwards R.R., Fillingim R.B., Ness T.J. (2003 Jan). Age-related differences in endogenous pain modulation: a comparison of diffuse noxious inhibitory controls in healthy older and younger adults. Pain.

[54] Potvin S., Grignon S., Marchand S. (2009 May). Human evidence of a supra-spinal modulating role of dopamine on pain perception. Synapse.

[55] Villanueva L., Chitour D., Le Bars D. (1986 Oct). Involvement of the dorsolateral funiculus in the descending spinal projections responsible for diffuse noxious inhibitory controls in the rat. J. Neurophysiol..

[56] Bouhassira D., Villanueva L., Bing Z., le Bars D. (1992 Nov 13). Involvement of the subnucleus reticularis dorsalis in diffuse noxious inhibitory controls in the rat. Brain Res..

[57] Le Bars D., Villanueva L., Bouhassira D., Willer J.C. (1992). Diffuse noxious inhibitory controls (DNIC) in animals and in man. Patol. Fiziol. Eksp. Ter..

[58] Bannister K., Kucharczyk M.W., Graven-Nielsen T., Porreca F. (2021 Jul 1). Introducing descending control of nociception: a measure of diffuse noxious inhibitory controls in conscious animals. Pain.

[59] Leite-Almeida H., Valle-Fernandes A., Almeida A. (2006 Jun 30). Brain projections from the medullary dorsal reticular nucleus: an anterograde and retrograde tracing study in the rat. Neuroscience.

[60] Kucharczyk M.W., Valiente D., Bannister K. (2021 Apr 20). Developments in understanding diffuse noxious inhibitory controls: pharmacological evidence from pre-clinical research. J. Pain Res..

[61] Häring M., Zeisel A., Hochgerner H., Rinwa P., Jakobsson J.E.T., Lönnerberg P., La Manno G., Sharma N., Borgius L., Kiehn O., Lagerström M.C., Linnarsson S., Ernfors P. (2018 Jun). Neuronal atlas of the dorsal horn defines its architecture and links sensory input to transcriptional cell types. Nat. Neurosci..

[62] Carta M., Carlsson T., Kirik D., Björklund A. (2007 Jul). Dopamine released from 5-HT terminals is the cause of L-DOPA-induced dyskinesia in parkinsonian rats. Brain.

[63] Dellapina E., Gerdelat-Mas A., Ory-Magne F., Pourcel L., Galitzky M., Calvas F., Simonetta-Moreau M., Thalamas C., Payoux P., Brefel-Courbon C. (2011 Jan). Apomorphine effect on pain threshold in Parkinson’s disease: a clinical and positron emission tomography study. Mov. Disord..

[64] Altier N., Stewart J. (1999). The role of dopamine in the nucleus accumbens in analgesia. Life Sci..

[65] Gear R.W., Aley K.O., Levine J.D. (1999 Aug 15). Pain-induced analgesia mediated by mesolimbic reward circuits. J. Neurosci..

[66] Fleetwood-Walker S.M., Hope P.J., Mitchell R. (1988 May). Antinociceptive actions of descending dopaminergic tracts on cat and rat dorsal horn somatosensory neurones. J. Physiol..

[67] Lapirot O., Melin C., Modolo A., Nicolas C., Messaoudi Y., Monconduit L., Artola A., Luccarini P., Dallel R. (2011 Aug). Tonic and phasic descending dopaminergic controls of nociceptive transmission in the medullary dorsal horn. Pain.

[68] Millan M.J. (2002 Apr). Descending control of pain. Prog. Neurobiol..

[69] Chaudhuri K.R., Rizos A., Trenkwalder C., Rascol O., Pal S., Martino D., Carroll C., Paviour D., Falup-Pecurariu C., Kessel B., Silverdale M., Todorova A., Sauerbier A., Odin P., Antonini A., Martinez-Martin P., EUROPAR and the IPMDS Non Motor PD Study Group (2015 Oct). King’s Parkinson’s disease pain scale, the first scale for pain in PD: an international validation. Mov. Disord..

[70] Ford B. (2010). Pain in Parkinson’s disease. Mov. Disord..

[71] Kofler M., Leis A.A., Valls-Solé J. (2019 Apr). Cutaneous silent periods - part 1: update on physiological mechanisms. Clin. Neurophysiol..

